# Predictors for development of oxaliplatin-induced peripheral neuropathy in cancer patients as determined by ordered logistic regression analysis

**DOI:** 10.1371/journal.pone.0275481

**Published:** 2022-09-29

**Authors:** Yuko Kanbayashi, Takeshi Ishikawa, Yoshiaki Kuriu, Eigo Otsuji, Koichi Takayama

**Affiliations:** 1 Department of Outpatient Oncology Unit, University Hospital, Kyoto Prefectural University of Medicine, Kyoto, Japan; 2 Department of Education and Research Center for Clinical Pharmacy, Faculty of Pharmacy, Osaka Medical and Pharmaceutical University, Takatsuki, Osaka, Japan; 3 Department of Molecular Gastroenterology and Hepatology, Graduate School of Medical Science, Kyoto Prefectural University of Medicine, Kyoto, Japan; 4 Division of Digestive Surgery, Department of Surgery, Graduate School of Medical Science, Kyoto Prefectural University of Medicine, Kyoto, Japan; 5 Department of Pulmonary Medicine, Graduate School of Medical Science, Kyoto Prefectural University of Medicine, Kyoto, Japan; University of Sydney, AUSTRALIA

## Abstract

**Background:**

Oxaliplatin causes acute cold-induced neurotoxicity and chronic cumulative neuropathy, which can require dose modification and impacts quality of life. However, effective strategies for managing oxaliplatin-induced peripheral neuropathy (OIPN) among affected patients remain elusive.

**Objective:**

This retrospective study aimed to identify predictors for the development of OIPN.

**Methods:**

Participants comprised 322 cancer patients at our hospital who were receiving oxaliplatin between January 2017 and March 2021. For the regression analysis of factors associated with OIPN, variables were manually extracted from medical charts. The severity of OIPN was evaluated using the National Cancer Institute’s Common Terminology Criteria for Adverse Events, version 5. Multivariate ordered logistic regression analysis was performed to identify predictors for the development of OIPN. Optimal cut-off thresholds were determined using receiver operating characteristic analysis. Values of *P* <0.05 (2-tailed) were considered significant.

**Results:**

Significant risk factors identified included higher body mass index (BMI) (odds ratio [OR] = 1.06, 95% confidence interval [CI] = 1.00–1.12; *P* = 0.043), female sex (OR = 1.67, 95%CI = 1.06–2.61; *P* = 0.026) and higher total dosage (OR = 2.39, 95%CI = 1.67–3.42; *P* = < 0.0001).

**Conclusion:**

High BMI, female sex and high total dosage were identified as significant predictors for the development of OIPN.

## Introduction

Oxaliplatin is a platinum-derivative chemotherapeutic agent used for patients with colorectal, pancreatic, or gastric cancer [1–3]. Oxaliplatin can cause acute cold-induced neurotoxicity and chronic cumulative neuropathy, requiring dose modification and impacting quality of life (QOL) [4–6]. Early identification of neurotoxicity and changes in dosage or dosing schedule could prevent the development of chronic symptoms, which, once established, may take many months or years to resolve, or may even persist throughout life with substantial detrimental effects on QOL. Various studies have statistically analyzed predictors for oxaliplatin-induced peripheral neuropathy (OIPN) [7–10]. Nevertheless, effective strategies for managing OIPN among affected patients remain elusive. Thus, in addition to improving QOL for patients undergoing chemotherapy, the need for methods to better identify patients at risk of OIPN remains unmet. This retrospective study was therefore undertaken to identify predictors associated with the development of OIPN to help guide future strategies toward improving safety, efficacy, and QOL among cancer patients treated with oxaliplatin.

## Patients and methods

### Study period and participants

This study retrospectively analyzed 340 cancer patients who received oxaliplatin at our hospital between January 2017 and March 2021. Only those patients who received only one cycle of oxaliplatin were excluded. The Medical Ethics Review Committee of Kyoto Prefectural University of Medicine approved this study (approval no. ERB-C-867-3). All procedures were performed in accordance with the ethical standards of the institutional medical ethics review committee at Kyoto Prefectural University of Medicine and the 1964 Declaration of Helsinki and its later amendments. No prospective studies with human participants or animals were performed by any of the authors in association with this article. Given the retrospective nature of this work, the need to obtain informed consent was waived for the individual participants included in the study, in accordance with the standards of the institutional medical ethics review committee at Kyoto Prefectural University of Medicine.

### Extraction of variables

For the regression analysis of factors associated with OIPN, variables were manually extracted from medical charts. Evaluated variables included factors that could potentially impact the development of OIPN based on previous studies [7–10] and clinical significance: demographic data (sex, age, height, weight, body surface area and body mass index [BMI]), type of cancer, number of cycles, logarithmic transformation of total dosage, concomitant medications (proton pump inhibitors [PPIs], renin-angiotensin system [RAS] inhibitors), analgesic adjuvant, chemotherapy regimen [FOLFOX: 5-fluorouracil, oxaliplatin (85 mg/m^2^) and leucovorin; SOX: S-1 plus oxaliplatin (100–130 mg/m^2^); XELOX: capecitabine plus oxaliplatin (130 mg/m^2^); or FOLFIRINOX: 5-fluorouracil, oxaliplatin (85 mg/m^2^), irinotecan and leucovorin. S-1 is an orally available chemotherapeutic agent comprising tegafur (a prodrug of fluorouracil [5-FU]), gimeracil (preventing dihydropyrimidine dehydrogenase-mediated degradation of 5-FU), and oteracil (reducing the toxic effects of 5-FU)], presence of comorbidities (diabetes mellitus), and laboratory test values. Creatinine clearance was estimated using the Cockcroft and Gault equation based on serum creatinine, sex, age, and weight. Clinical information was extracted from before administration of the first dose of oxaliplatin. Concomitant medication was defined as administration of another drug for ≥2 weeks as of the time of evaluation. In this study, all symptoms including OIPN were recorded using a questionnaire that has been used for all patients in our outpatient oncology center since 2012. Information on the severity of OIPN was provided by each patient in our center with the help of our qualified nurses. The questionnaire was made according to the National Cancer Institute Common Toxicity Criteria for Adverse Events (NCI-CTCAE; version 5) after every cycle of chemotherapy. Information on the severity of OIPN was extracted at the time of final oxaliplatin administration.

### Statistical analysis

Independent variables were analyzed for multicollinearity (correlation coefficient |r| ≥0.7), since correlations among variables can lead to unreliable and unstable results from regression analyses. First, univariate ordered logistic regression analyses between outcomes and each potential independent variable were performed. Subsequently, a multivariate ordered logistic regression model was constructed by employing those candidate variables showing values of *P* <0.05 from univariate regression or selected based on previous studies [7–10]. Multivariate ordered logistic regression analysis was employed because the severity of OIPN was evaluated using a graded scale and multiple factors actually associated as predictors for the development of OIPN had to be analyzed concurrently. Optimal cut-off thresholds were determined using receiver operating characteristic (ROC) curve analysis. Furthermore, whether any difference existed in the development of OIPN among cancer types was analyzed. The Wilcoxon/Kruskal-Wallis test was used to identify significant difference between groups.

For all statistical analyses, values of *P* <0.05 (two-tailed) were considered significant. All statistical analyses were performed using JMP Pro^®^ version 16.1 (SAS Institute, Cary, NC, USA).

## Results

Of the 340 patients who received oxaliplatin, all 18 patients who discontinued oxaliplatin after only one cycle were excluded. Table 1 presents the clinical characteristics of the 322 enrolled patients, the potential variables related to the development of OIPN, and the results of univariate analyses.

**Table 1:** Patient characteristics, extracted variables, and results of univariate analyses (n = 322).

	Grade 0 (n = 65)	Grade 1 (n = 165)	Grade 2 (n = 69)	Grade 3 (n = 23)	*P* value	Odds ratio (95%CI)
**Demographic data**						
Male, n (%)	50	105	38	14	**0.015**	0.58
	(76.9)	(63.6)	(55.1)	(60.9)		(0.38–0.90)
Female, n (%)	15	60	31	9	**0.015**	1.71
	(23.1)	(36.4)	(44.9)	(39.1)		(1.11–2.64)
Age (y), median (range)	69	70	68	67	0.279	0.99
	(46–88)	(32–90)	(39–86)	(27–83)		(0.97–1.01)
Height (cm), median (range)	164.8	163.5	165.1	164.5	0.097	0.98
	(147.5–189.2)	(145–184.8)	(142–177.6)	(145–176)		(0.96–1.00)
Weight (kg), median (range)	54.8	55.3	55.6	59	0.598	1.00
	(28.4–94)	(31.1–89)	(31.2–103)	(39.3–80)		(0.99–1.10)
BMI (kg/m^2^), median (range)	20.6	20.8	20.8	21.9	0.117	1.04
	(12.2–31.2)	(12.7–30.7)	(13.2–33.7)	(16.1–28.4)		(0.99–1.10)
BMI (kg/m^2^) ≥ 25, n (%)	5	20	12	3	0.134	1.61
	(7.7)	(12.1)	(17.4)	(13.0)		(0.86–2.99)
BMI (kg/m2) ≥ 20.5, n (%)	33	87	38	19	0.077	1.45
	(50.8)	(52.7)	(55.1)	(82.6)		(0.96–2.21)
BSA (m^2^), median (range)	1.59	1.59	1.59	1.63	0.877	0.92
	(1.14–2.08)	(1.16–2.09)	(1.13–2.20)	(1.33–1.90)		(0.30–2.79)
**Cancer type**						
Gastric, n (%)	28	54	18	6	**0.032**	0.62
	(43.1)	(32.7)	(26.1)	(26.1)		(0.40–0.96)
Pancreas, n (%)	5	18	5	2	0.899	0.96
	(7.7)	(10.9)	(7.2)	(8.7)		(0.47–1.94)
Colorectal, n (%)	32	89	45	14	**0.049**	1.52
	(49.2)	(53.9)	(65.2)	(60.9)		(1.001–2.31)
**Comorbidity**						
Diabetes mellitus, n (%)	12	24	8	8	0.763	1.09
	(18.5)	(14.5)	(11.6)	(34.8)		(0.62–1.90)
**Laboratory test value before administration**						
Serum creatinine, mg/dL, median (range)	0.77	0.73	0.64	0.71	0.446	0.69
	(0.35–1.2)	(0.36–1.92)	(0.41–1.77)	(0.36–1.27)		(0.26–1.80)
Creatinine clearance, mL/min, median (range)	74.9	70.1	71.4	82.6	0.178	1.01
	(28.2–127.4)	(34.1–204.7)	(25–168.1)	(37.4–119.9)		(0.998–1.013)
**Number of cycles, median (range)**	5	8	9	8	**< .0001**	1.10
	(1–32)	(1–34)	(2–31)	(1–17)		(1.05–1.14)
**Total dosage, mg, median (range)**	595	910	1020	1040	**< .0001**	1.0007
	(130–4160)	(130–4420)	(260–2990)	(85–1040)		(1.0004–1.0011)
**Total dosage, mg, average (range)**	708	947	1108	1009	**< .0001**	1.0007
	(130–4160)	(130–4420)	(260–2990)	(85–1040)		(1.0004–1.0011)
**Logarithm (Total dosage, mg), median (range)**	6.39	6.81	6.93	6.95	**< .0001**	2.39
	(4.87–8.33)	(4.87–8.39)	(5.56–8.00)	(4.44–7.51)		(1.69–3.38)
**Regimen**						
FOLFOX	8	25	20	5	**0.014**	1.96
	(12.3)	(15.2)	(29.0)	(21.7)		(1.15–3.34)
SOX	27	45	11	5	**0.001**	0.46
	(41.5)	(27.3)	(15.9)	(21.7)		(0.28–0.73)
XELOX	22	70	30	7	0.624	1.11
	(33.8)	(42.4)	(43.5)	(30.4)		(0.73–1.69)
FOLFIRINOX	4	18	6	2	0.729	1.13
	(6.2)	(10.9)	(8.7)	(8.7)		(0.56–2.30)
**History of treatment with anticancer drugs**						
Cisplatin	0	5	3	0	0.390	1.77
		(3.0)	(8.7)			(0.48–6.52)
Taxane	1	8	3	2	0.245	1.81
	(1.5)	(4.8)	(4.3)	(8.7)		(0.67–4.89)
Oxaliplatin	0	2	3	0	0.206	2.86
		(1.2)	(4.3)			(0.56–14.52)
**Concomitant medication**						
RAS inhibitors	12	19	7	3	0.202	0.67
	(18.5)	(11.5)	(10.1)	(13.0)		(0.36–1.24)
Proton pump inhibitor	28	82	32	13	0.473	1.16
	(43.1)	(49.7)	(46.4)	(56.5)		(0.77–1.75)
Analgesic adjuvant	2	3	3	4	**0.009**	4.19
	(3.1)	(1.8)	(4.3)	(17.4)		(1.44–12.19)
*Duloxetine*	1	2	1	4	**0.002**	8.11
	(1.5)	(1.2)	(1.4)	(17.4)		(2.17–30.27)
*Gabapentinoids (Pregabalin or mirogabalin)*	1	2	2	0	0.798	1.24
	(1.5)	(1.2)	(2.9)			(0.24–6.50)
NSAIDs	4	17	8	1	0.623	1.19
	(6.2)	(10.3)	(11.6)	(4.3)		(0.59–2.42)
Opioids	8	15	14	1	0.455	1.27
	(12.3)	(9.1)	(20.3)	(4.3)		(0.67–2.40)

CI, confidence interval; BMI, body mass index; BSA, body surface area; FOLFOX, chemotherapy regimen consisting of fluorouracil plus oxaliplatin; SOX, chemotherapy regimen consisting of tegafur gimeracil oteracil potassium capsule (S-1) plus oxaliplatin; XELOX, chemotherapy regimen consisting of capecitabine plus oxaliplatin; FOLFIRINOX, chemotherapy regimen consisting of oxaliplatin, irinotecan, fluorouracil, and leucovorin; RAS, renin-angiotensin system; NSAIDs, non-steroidal anti-inflammatory drugs.

The selection of factors showing results of *P* <0.05 in univariate regression or based on previous studies identified the following candidate variables: female sex, higher BMI, higher logarithmic transformation of total dosage, use of RAS inhibitors, and use of PPIs.

Multivariate ordered logistic regression analysis was performed using these variables. Here, before the factors to be used in the multivariate analysis were selected, the analysis was conducted to check for multicollinearity between each factor. As men are typically larger than women, sex and BMI were predicted to be strongly correlated. However, the correlation coefficient between BMI and sex was r = 0.13, with no evidence of multicollinearity. On the other hand, in univariate analyses, gastric cancer, colorectal cancer, and FOLFOX and SOX regimens were factors significantly associated with OIPN. Odds ratios (ORs) indicated that patients with gastric cancer were less likely to develop OIPN and those with colorectal cancer were more likely to develop OIPN. However, the results of multicollinearity analysis showed strong correlations between gastric cancer and SOX regimen (r = 0.74), and between gastric cancer and colorectal cancer (r = -0.79). Disease and regimen were thus excluded from the explanatory variables used in the multivariate analysis because of the strong multicollinearity and because these factors affected the total dose.

Significant factors identified included higher BMI (OR = 1.06, 95% confidence interval [CI] = 1.00–1.12; *P* = 0.043), female sex (OR = 1.67, 95%CI = 1.06–2.61; *P* = 0.026) and higher logarithmic transformation of total dosage (OR = 2.39, 95%CI = 1.67–3.42; *P* = <0.0001) (Table 2).

**Table 2:** Results of multivariate ordered logistic regression analysis for variables extracted by forward selection (n = 322).

Variable	*P* value	Odds ratio	95%CI	
			Lower 95%	Upper 95%
RAS inhibitors	0.450	0.78	0.40	1.50
BMI	**0.043**	1.06	1.002	1.122
Proton pump inhibitors	0.063	1.50	0.98	2.31
Female	**0.026**	1.67	1.06	2.61
Age	0.766	1.00	0.98	1.02
Logarithm (total dosage)	**< .0001**	2.39	1.67	3.42

CI, confidence interval; RAS, renin-angiotensin system; BMI, body mass index

ROC analysis revealed that OIPN of grade 2 or higher was more likely among patients with BMI ≥20.5 kg/m^2^, with 63.3% sensitivity and 47.8% specificity (area under the curve [AUC] = 0.54). OIPN of grade 2 or higher was also more likely to occur with total dosage ≥680 mg, with 77.2% sensitivity and 44.8% specificity (AUC = 0.63), while OIPN of grade 3 or higher was more likely with total dose ≥1040 mg, with 60.9% sensitivity and 61.2% specificity (AUC = 0.59).

No significant difference in the development of OIPN was evident among cancer types (colon, gastric, and pancreas cancers; *P* = 0.4438).

## Discussion

The multivariate ordered logistic regression analysis performed in this study showed that significant predictors for the development of OIPN included higher BMI, female sex, and total dosage. Although concomitant use of RAS inhibitors was not extracted as a significant variable in multivariate analysis, the combined use of RAS inhibitors tended to reduce OIPN. In contrast, concomitant use of PPIs was suggested to represent a risk factor for OIPN.

In this study, BMI was extracted as a significant predictor for the development of OIPN. In previous studies, obesity has been reported as a risk factor for chemotherapy-induced peripheral neuropathy [7, 11–13]. In addition, when the body fat content is high, anticancer drugs accumulate in adipose tissue and excretion is thus delayed [14]. Notably, however, ROC curve analysis revealed a BMI cut-off of 20.5 kg/m^2^ for the group likely to develop OIPN (grade 2 or higher). The World Health Organization defines individuals with BMI ≥25 kg/m^2^ as obese or overweight [15]. Despite that, in this study, OIPN was more likely to occur with BMI as low as 20.5 kg/m^2^. This may be due to differences in physique between Japanese and Western populations. Clinicians should pay close attention to the onset of OIPN among patients with BMI ≥20.5 kg/m^2^, and not necessarily just the obese population. Our results only suggest that the higher the BMI, the more likely OIPN is to occur. Further investigation of these issues in different populations is warranted.

In this study, women were suggested to be more likely to develop OIPN. Chemotherapy-induced peripheral neuropathy is sometimes associated with neuropathic pain. A number of reports have examined sex differences in pain sensitivity [16–24], with many concluding that women more frequently report pain for many chronic pain types, including cancer pain [7, 18–23]. The results of the present study were consistent with the findings of those previous studies. In a study examining differences between women and men in the way they experience cold-pressor pain, Keogh et al. reported that women who concentrated on the emotional aspects of pain may actually experience more pain as a result, possibly because the emotions associated with pain were negative [20–22]. Hau et al. found that testosterone reduced responsiveness to nociceptive stimuli in wild birds [23]. They demonstrated that analgesic drugs such as morphine activated G protein-sensitive inwardly rectifying potassium (GIRK)2 [23]. Thus, men may have a higher threshold of tolerable pain because GIRK2 has been shown to be present at higher levels in men than in women. Conversely, other reports have found no sex differences in pain or that men are more prone to experiencing some types of chronic pain, including cancer pain [17, 24]. Further verification of sex differences in painful chemotherapy-induced peripheral neuropathy is therefore needed.

Our results also showed that OIPN was more likely to develop as total dosage increased. ROC analysis revealed that OIPN of grade 2 or higher was more likely to occur with a dose ≥680 mg, and OIPN of grade 3 or higher with a dose of ≥1040 mg. Regarding total dosage, OIPN tends to become chronic depending on the total dosage [5, 8–10, 25]. Previous studies have reported that with FOLFOX therapy, the median cumulative oxaliplatin dose at which Grade 2 or 3 peripheral neuropathy occurs is about 850 mg/m^2^ [6]. Beijers et al. also demonstrated that a higher cumulative dose is associated with the development of long-term OIPN [8]. Careful attention should be paid to the cumulative dose of oxaliplatin, particularly administration of doses ≥680 mg.

In univariate analysis, gastric cancer, colorectal cancer, and FOLFOX and SOX regimens were factors significantly associated with OIPN. ORs indicated that patients with gastric cancer were less likely to develop OIPN, while those with colorectal cancer were more likely to develop OIPN. On the other hand, patients receiving the SOX regimen were less likely to develop OIPN and those on the FOLFOX regimen were more likely to develop OIPN. Gastric cancer correlated strongly with the SOX regimen, which is primarily administered as a 3-week regimen for gastric cancer. Similarly, FOLFOX is given every 2 weeks for colorectal cancer. The longer oxaliplatin dosing interval may have prevented the development of OIPN. Further investigation of these issues is needed.

Concomitant use of PPI was not extracted as a significant predictor for the development of OIPN in the present study, but has been suggested as a risk factor for chemotherapy—induced peripheral neuropathy. Previous studies have reported PPI use as a risk factor for peripheral neuropathy [26–28]. Makunts et al. found a significant increase in a wide variety of peripheral neurological and neuropathic adverse events due to PPI [26]. They reported that an increased gastric pH level correlated with decreased levels of vitamin B12. In turn, B12 deficiency has been associated with reversible peripheral neuropathy [28]. Clinicians should thus be careful in prescribing PPIs when performing chemotherapy with oxaliplatin.

Although not significant, concomitant use of RAS inhibitors has been suggested to prevent OIPN. Previous research has reported RAS inhibitors could prevent OIPN [29]. Further research on this point is needed.

Several limitations to the current study need to be considered. First, the retrospective nature of the study may have decreased the validity of the data obtained. Second, since this study was performed at a single institute, prospective multicenter studies are needed to confirm the results. Third, the possibility of confounding, selection, and information biases cannot be fully excluded in this study. That is, full information about the participants (e.g., general information regarding behaviors and status of living) was not available for consideration.

In conclusion, higher BMI, female sex, and higher total dosage were identified as significant risk factors for the development of OIPN. While our findings need to be confirmed in further studies, these results may assist in developing strategies to improve QOL among patients receiving oxaliplatin.

## References

[1] Qin S, Li J, Wang L, Xu J, Cheng Y, Bai Y, et al. Efficacy and Tolerability of First-Line Cetuximab Plus Leucovorin, Fluorouracil, and Oxaliplatin (FOLFOX-4) Versus FOLFOX-4 in Patients With RAS Wild-Type Metastatic Colorectal Cancer: The Open-Label, Randomized, Phase III TAILOR Trial. J Clin Oncol. 2018; 36:3031–3039. doi: 10.1200/JCO.2018.78.3183 30199311 PMC6324088

[2] Boku N, Ryu MH, Kato K, Chung HC, Minashi K, Lee KW, et al. Safety and efficacy of nivolumab in combination with S-1/capecitabine plus oxaliplatin in patients with previously untreated, unresectable, advanced, or recurrent gastric/gastroesophageal junction cancer: interim results of a randomized, phase II trial (ATTRACTION-4). Ann Oncol. 2019; 30:250–258. doi: 10.1093/annonc/mdy540 30566590 PMC6386029

[3] Conroy T, Hammel P, Hebbar M, Ben Abdelghani M, Wei AC, Raoul JL, et al. FOLFIRINOX or Gemcitabine as Adjuvant Therapy for Pancreatic Cancer. N Engl J Med. 2018; 379:2395–2406. doi: 10.1056/NEJMoa1809775 30575490

[4] Cavaletti G, Marmiroli P. Management of Oxaliplatin-Induced Peripheral Sensory Neuropathy. Cancers (Basel). 2020; 12:1370. doi: 10.3390/cancers12061370 32471028 PMC7352541

[5] Wei G, Gu Z, Gu J, Yu J, Huang X, Qin F, et al. Platinum accumulation in oxaliplatin-induced peripheral neuropathy. J Peripher Nerv Syst. 2021; 26:35–42. doi: 10.1111/jns.12432 33462873 PMC7986112

[6] Kang L, Tian Y, Xu S, Chen H. Oxaliplatin-induced peripheral neuropathy: clinical features, mechanisms, prevention and treatment. J Neurol. 2021; 268:3269–3282. doi: 10.1007/s00415-020-09942-w 32474658

[7] Mizrahi D, Park SB, Li T, Timmins HC, Trinh T, Au K, et al. Hemoglobin, Body Mass Index, and Age as Risk Factors for Paclitaxel- and Oxaliplatin-Induced Peripheral Neuropathy. JAMA Netw Open. 2021 4: e2036695. doi: 10.1001/jamanetworkopen.2020.36695 33587134 PMC7885037

[8] Beijers AJ, Mols F, Tjan-Heijnen VC, Faber CG, van de Poll-Franse LV, Vreugdenhil G. Peripheral neuropathy in colorectal cancer survivors: the influence of oxaliplatin administration. Results from the population-based PROFILES registry. Acta Oncol. 2015; 54:463–9. doi: 10.3109/0284186X.2014.980912 . 25417732

[9] Kanbayashi Y, Hosokawa T, Okamoto K, Konishi H, Otsuji E, Yoshikawa T, et al. Statistical identification of predictors for peripheral neuropathy associated with administration of bortezomib, taxanes, oxaliplatin or vincristine using ordered logistic regression analysis. Anticancer Drugs. 2010; 21:877–81. doi: 10.1097/CAD.0b013e32833db89d 20679888

[10] Hsu SY, Huang WS, Lee SH, Chu TP, Lin YC, Lu CH, et al. Incidence, severity, longitudinal trends and predictors of acute and chronic oxaliplatin-induced peripheral neuropathy in Taiwanese patients with colorectal cancer. Eur J Cancer Care (Engl). 2019; 28: e12976. doi: 10.1111/ecc.12976 . 30536809

[11] Cox-Martin E, Trahan LH, Cox MG, Dougherty PM, Lai EA, Novy DM. Disease burden and pain in obese cancer patients with chemotherapy-induced peripheral neuropathy. Support Care Cancer. 2017; 25:1873–1879. doi: 10.1007/s00520-017-3571-5 28124735 PMC5439217

[12] Petrovchich I, Kober KM, Wagner L, Paul SM, Abrams G, Chesney MA, et al. Deleterious Effects of Higher Body Mass Index on Subjective and Objective Measures of Chemotherapy-Induced Peripheral Neuropathy in Cancer Survivors. J Pain Symptom Manage. 2019; 58:252–263. doi: 10.1016/j.jpainsymman.2019.04.029 31047960 PMC6679783

[13] Sajdyk TJ, Boyle FA, Foran KS, Tong Y, Pandya P, Smith EML, et al. Obesity as a Potential Risk Factor for Vincristine-induced Peripheral Neuropathy. J Pediatr Hematol Oncol. 2020;42: e637–e640. doi: 10.1097/MPH.0000000000001604 31634238 PMC7165027

[14] Blouin RA, Warren GW. Pharmacokinetic considerations in obesity. J Pharm Sci. 1999; 88:1–7. doi: 10.1021/js980173a 9874695

[15] World Health Organization. Body Mass Index—BMI. 2022. (https://www.euro.who.int/en/health-topics/disease-prevention/nutrition/a-healthy-lifestyle/body-mass-index-bmi). Accessed March 25, (2022).

[16] Won A, Lapane K, Gambassi G, Bernabei R, Mor V, Lipsitz LA. Correlates and management of nonmalignant pain in the nursing home. SAGE Study Group. Systematic Assessment of Geriatric drug use via Epidemiology. J Am Geriatr Soc. 1999; 47:936–42. doi: 10.1111/j.1532-5415.1999.tb01287.x . 10443853

[17] Gear RW, Miaskowski C, Gordon NC, Paul SM, Heller PH, Levine JD. Kappa-opioids produce significantly greater analgesia in women than in men. Nat Med. 1996; 2:1248–50. doi: 10.1038/nm1196-1248 . 8898754

[18] Unruh AM. Gender variations in clinical pain experience. Pain. 1996; 65:123–67. doi: 10.1016/0304-3959(95)00214-6 . 8826503

[19] Fillingim RB. Sex, gender, and pain: women and men really are different. Curr Rev Pain. 2000; 4:24–30. doi: 10.1007/s11916-000-0006-6 . 10998712

[20] Keogh E, Bond FW, Hanmer R, Tilston J. Comparing acceptance- and control-based coping instructions on the cold-pressor pain experiences of healthy men and women. Eur J Pain. 2005; 9:591–8. doi: 10.1016/j.ejpain.2004.12.005 . 16139188

[21] Keogh E, Mounce C, Brosnan M. Can a sexually dimorphic index of prenatal hormonal exposure be used to examine cold pressor pain perception in men and women? Eur J Pain. 2007; 11:231–6. doi: 10.1016/j.ejpain.2006.02.011 . 16603392

[22] Kowalczyk WJ, Evans SM, Bisaga AM, Sullivan MA, Comer SD. Sex differences and hormonal influences on response to cold pressor pain in humans. J Pain. 2006; 7:151–60. doi: 10.1016/j.jpain.2005.10.004 . 16516820

[23] Hau M, Dominguez OA, Evrard HC. Testosterone reduces responsiveness to nociceptive stimuli in a wild bird. Horm Behav. 2004; 46:165–70. doi: 10.1016/j.yhbeh.2004.02.007 . 15256306

[24] Kanbayashi Y, Okamoto K, Ogaru T, Hosokawa T, Takagi T. Statistical validation of the relationships of cancer pain relief with various factors using ordered logistic regression analysis. Clin J Pain. 2009; 25:65–72. doi: 10.1097/AJP.0b013e31817e1379 . 19158548

[25] Yokoyama S, Nakagawa C, Hosomi K. Treatment strategy of oxaliplatin-induced peripheral neuropathy: a retrospective, nationwide study. Support Care Cancer. 2022 Feb;30(2):1765–1773. doi: 10.1007/s00520-021-06585-z Epub 2021 Oct 1. . 34595605

[26] Makunts T, Alpatty S, Lee KC, Atayee RS, Abagyan R. Proton-pump inhibitor use is associated with a broad spectrum of neurological adverse events including impaired hearing, vision, and memory. Sci Rep. 2019; 9:17280. doi: 10.1038/s41598-019-53622-3 31754136 PMC6872761

[27] Rajabally YA, Jacob S. Neuropathy associated with lansoprazole treatment. Muscle Nerve. 2005; 31:124–5. doi: 10.1002/mus.20155 . 15468341

[28] Heidelbaugh JJ. Proton pump inhibitors and risk of vitamin and mineral deficiency: evidence and clinical implications. Ther Adv Drug Saf. 2013; 4:125–33. doi: 10.1177/2042098613482484 25083257 PMC4110863

[29] Uchida M, Kawazoe H, Takatori S, Namba H, Uozumi R, Tanaka A, et al. Preventive Effects of Renin-angiotensin System Inhibitors on Oxaliplatin-induced Peripheral Neuropathy: A Retrospective Observational Study. Clin Ther. 2018; 40:1214–1222.e1. doi: 10.1016/j.clinthera.2018.05.011 29983264

